# Even Visually Intact Cell Walls in Waterlogged Archaeological Wood Are Chemically Deteriorated and Mechanically Fragile: A Case of a 170 Year-Old Shipwreck

**DOI:** 10.3390/molecules25051113

**Published:** 2020-03-03

**Authors:** Liuyang Han, Xingling Tian, Tobias Keplinger, Haibin Zhou, Ren Li, Kirsi Svedström, Ingo Burgert, Yafang Yin, Juan Guo

**Affiliations:** 1 Department of Wood Anatomy and Utilization, Research Institute of Wood Industry, Chinese Academy of Forestry, Beijing 100091, China; 2 Wood Collections (WOODPEDIA), Chinese Academy of Forestry, Beijing 100091, China; 3 Heritage Conservation and Restoration Institute, Chinese Academy of Cultural Heritage, Beijing 100029, China; 4 Wood Materials Science, ETH Zürich, 8093 Zürich, Switzerland; 5 Laboratory for Cellulose & Wood Materials, EMPA, 8600 Dübendorf, Switzerland; 6 Pilot Base, Chinese Academy of Forestry, Beijing 102300, China; 7 Department of Physics, University of Helsinki, FI-00014 Helsinki, Finland

**Keywords:** waterlogged archaeological wood, cell wall structure, cell wall mechanics, deterioration, FTIR imaging, Raman imaging

## Abstract

Structural and chemical deterioration and its impact on cell wall mechanics were investigated for visually intact cell walls (VICWs) in waterlogged archaeological wood (WAW). Cell wall mechanical properties were examined by nanoindentation without prior embedding. WAW showed more than 25% decrease of both hardness and elastic modulus. Changes of cell wall composition, cellulose crystallite structure and porosity were investigated by ATR-FTIR imaging, Raman imaging, wet chemistry, ^13^C-solid state NMR, pyrolysis-GC/MS, wide angle X-ray scattering, and N_2_ nitrogen adsorption. VICWs in WAW possessed a cleavage of carboxyl in side chains of xylan, a serious loss of polysaccharides, and a partial breakage of β-O-4 interlinks in lignin. This was accompanied by a higher amount of mesopores in cell walls. Even VICWs in WAW were severely deteriorated at the nanoscale with impact on mechanics, which has strong implications for the conservation of archaeological shipwrecks.

## 1. Introduction

Wood is one of the most widely distributed natural macromolecular materials in the world and is also highly degradable though it is widely applied. A large number of waterlogged archaeological wooden artifacts have been excavated all over the world [1,2], among which shipwrecks provide unique insight into the science, technology and inheriting historical culture of their time. The conservation of WAW is of great importance, but very challenging and complex. Cell walls of archaeological wood deteriorate to a certain extent during their long-term burial [1,3], causing significant changes in cellulose crystallites [4,5], porosity [6], morphological structure [7] and mechanical properties [8]. Therefore, WAW is at high risk of being damaged upon ineluctable water-removal, which is necessary to avoid further deterioration by microorganisms, for archaeological studies and for public exhibition. The risk of damage mainly originates from impaired mechanical properties of wood cell walls, which makes WAW more prone to failure under drying stresses as compared with reference wood (RW) during dehydration [9]. Although the mechanical properties of cell walls in WAW play a key role for the resistance against collapse and fracture during water-removal treatments, only few respective reports have been published, due to the difficulties in sample preparation and the potential influence of applied embedding agents [10].

WAW is usually divided into severely, moderately and slightly deteriorated wood according to the maximum water contents (MWC) of the WAW [8,11,12]. Cell walls in these deterioration states can be easily differentiated into VICWs and decayed cell walls based on visible cell wall erosion [13,14]. Wood cell wall mechanics are highly dependent on the chemical composition and cell wall structure, including porosity [15,16], thus cell wall alteration in deterioration processes should be reflected in the decrease of mechanical properties of cell walls in WAW. One can assume that wood cells with VICWs possess higher mechanical properties than wood cells with eroded cell walls because they have more remaining cell wall material. Considering the difficulty of testing the mechanical properties of decayed cell walls and the large variability, here we focus on the analysis of VICWs to gain insight into the preservation state of the WAW. The obtained data can be used as a basis for a decision on which consolidation measures are advised for shipwreck restauration, e.g., wood drying without consolidation or pretreatments with specific consolidates. Previous articles reported severe chemical alterations of WAW including the elimination of acetyl side chains and the cleavage of backbones in hemicelluloses, the depolymerization of amorphous cellulose, and the partial depletion of β-O-4 links as well as the modification of functional groups in lignin [2,17]. The deterioration of cell wall components further results in a higher porosity and a decrease of crystallinity [4,5]. However, related research on VICWs in WAW is scarce.

The aim of this research was to investigate the effect of structural and chemical deterioration on micromechanics of VICWs in WAW, in order to provide scientific knowledge for the selection of the right water-removal strategy and a choice of potential consolidates for conservation treatments of WAW. We collected waterlogged archaeological hardwood (*Hopea* sp.) from the marine “Xiaobaijiao No. 1” shipwreck dated from 1821–1850 AD. VICWs in WAW were investigated at the cell level, including scanning electron microscopy (SEM), nanoindentation (NI) without embedding, attenuated total reflection Fourier-transform infrared (ATR-FTIR) imaging and Raman imaging. Composition, cellulose crystallite structure and porosity of WAW cell walls were studied by wet chemistry, ^13^C-solid state NMR, pyrolysis-GC/MS, wide angle X-ray scattering (WAXS) and N_2_ nitrogen adsorption.

## 2. Results and Discussion

### 2.1. Structure and Mechanics of VICWs

**Figure 1:**
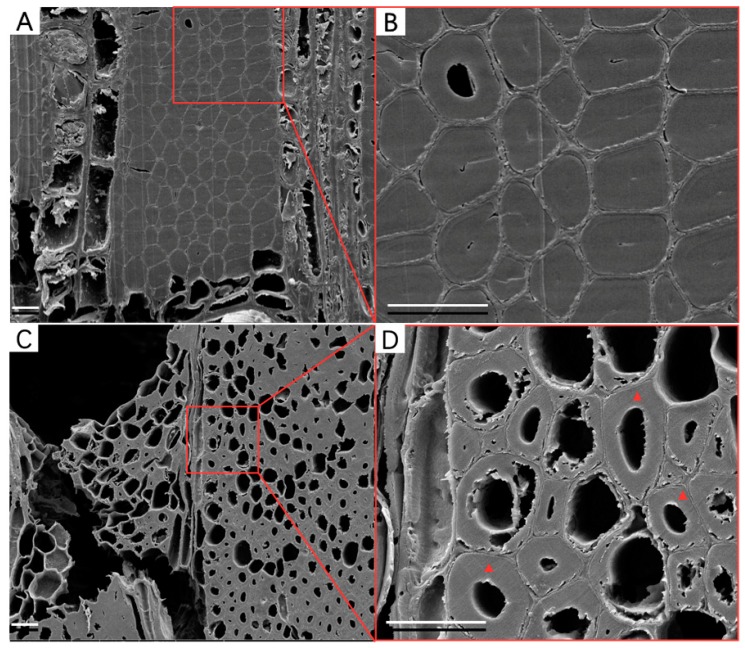
SEM images taken from the cross section of RW (**A**,**B**) and WAW (**C**,**D**). Scale bar = 20 µm. The observed cell walls decayed from S_3_ layer to S_1_ layer (D), which is probably the decay pattern of erosion bacteria. VICWs (D, red triangles) were the research objectives for NI, ATR-FTIR imaging and Raman imaging.

SEM analysis clearly revealed that WAW contained different deterioration states, whereas RW did not show any signs of deterioration (Figure 1). Although the waterlogged environment can prevent wood deterioration to a certain extent, wood cell walls are degraded due to the influence of local environment factors such as bacteria, fungi, acid and alkali [8]. Characteristic for WAW is an inhomogeneous deterioration pattern, even at the microscale. As shown in Figure 1B,D and Figure S1, the VICWs in WAW presented a similar intact morphology as cell walls in RW. There was no obvious detachment of secondary cell walls from the middle lamella and no larger visible pores (Figure 1D, red triangles).

**Figure 2:**
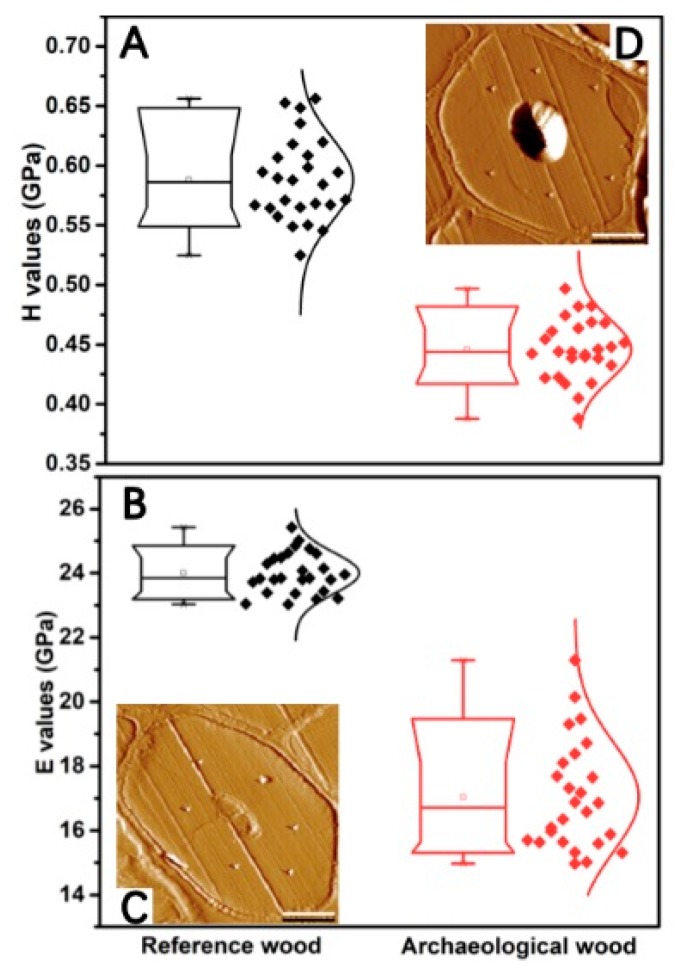
Boxplots of hardness (**A**) and indentation modulus (**B**) of S_2_ layer in wood fibers of RW (the black dots) and VICWs in archaeological wood (the red dots). Representative AFM images of cell walls in RW (**C**) and archaeological wood (**D**). Scale bar = 5 µm.

The mechanics of VICWs of WAW was analyzed using nanoindentation without embedding, which can avoid artifacts caused by the embedding medium [18] and provides more accurate mechanical properties of VICWs in WAW. The main function of hardwood fibers is mechanical support, which is mainly based on the thick second layer (S_2_ layer), which is thicker than the first layer (S_1_ layer) and the third layer (S_3_ layer) of secondary cell walls (Figure 2C,D). The S_2_ layer in fibers has a significant influence on the longitudinal mechanical properties of wood [18,19].

As shown in Figure 2, the hardness of S_2_ layer in fibers of RW and VICWs in archaeological wood was 0.59 GPa, with a coefficient of variation (COV) of 5.92% and 0.45 GPa with a COV of 5.66%, respectively. Their indentation moduli were 23.99 GPa with a COV of 2.66% and 17.04 GPa with a COV of 9.94%, respectively. Both the hardness and indentation moduli of VICWs in archaeological wood were significantly lower than those of RW (*p* < 0.05). These results are remarkable, as despite the unaltered morphological structure of VICWs in archaeological wood, they show a more than 25% decrease in hardness and indentation modulus, compared to RW.

Even though the morphologies of VICWs in archaeological wood were relatively well preserved, they were weakened in both the stiffness and hardness. According to previous studies, alteration of mechanics of cell walls from archaeological wood may result from the degradation of cell wall components [20,21]. Therefore, it can be assumed that the alteration of mechanical properties may be caused by changes in chemical composition of the cell walls, the cellulose crystallites structure as well as the porosity after the long-term deterioration, which are analyzed in detail in the following.

### 2.2. Cell Wall Composition of VICWs

#### 2.2.1. The Deterioration of Cell Wall Components

ATR-FTIR imaging with the spatial resolution of 1.56 μm and Raman imaging with the spatial resolution of ~0.3 μm were used to investigate the compositional changes in VICWs of archaeological wood. As shown in Figure S2A, the VICWs in archaeological wood can be preponderantly separated from the RW. Variations in the spectra of wood specimens were found in PC1 (31%), which captures the most variation in principal compositional analysis (PCA). The PC1 loading showed negative high absorbance from bands at 1730 cm^−1^, 1502 cm^−1^, 1370 cm^−1^, 1234 cm^−1^, and 834 cm^−1^ (Figure S2B), which are mainly ascribed to the C=O stretch in the glucuronic acid of O-acetyl-(4-O-methylgulcurono) xylan, the aromatic skeletal vibration in the lignin, the C–H bending in cellulose, the C–O stretching in the O=C–O group of side chains in hemicelluloses [22] and the C–H out of plane deformation at positions 2 and 6 of the syringyl units in lignin (Table S1). The negative PC1 loading correlates with the negative PC1 scores for VICWs in archaeological wood and with positive PC1 scores for the cell walls in RW. This indicates that the VICWs in archaeological wood are characterized by the loss of carboxyl groups in glucuronic acids of O-acetyl-(4-O-methylgulcurono) xylan as well as by the degradation of cellulose. The 26% and 68% decrease of glucose and xylose for the WAW compared to RW, shown by the wet chemical analysis (Table 1), also confirmed the degradation of polysaccharides.

**Figure 3:**
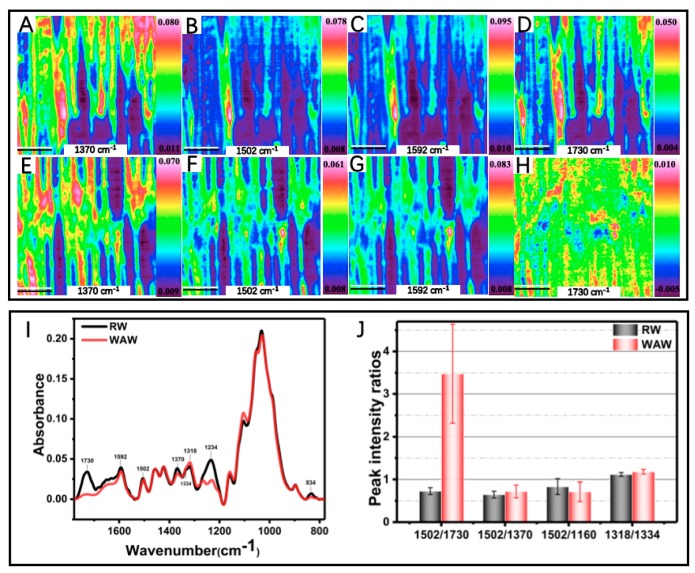
Pseudo-color FTIR spectral images of RW (**A**–**D**) and VICWs in archaeological wood (**E**–**H**). (**A**,**E**) spectral images generated using the band at 1370 cm^−1^, (**B**,**F**) Spectral images generated using the band at 1502 cm^−1^, (**C**,**G**) spectral images generated using the band at 1592cm^−1^, (**D**,**H**) spectral images generated using the band at 1730 cm^−1^. Scale bar = 50 µm. (**I**) Average FTIR absorbance spectra of RW (the black line) and VICWs in archaeological wood (the red line). (**J**) Histograms of peak intensity ratios of selected bands between RW and archaeological wood.

**Figure 4:**
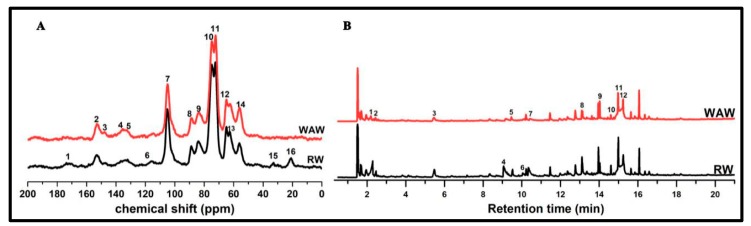
NMR spectra (**A**) and Py–GC/MS spectra (**B**) of *Hopea* wood.

Figure 3I shows the respective average FTIR spectra. In accordance with the PCA results, strong decreases of intensities for band peaks at 1730 cm^−1^, 1234 cm^−1^ and 834 cm^−1^ were observed in the average FTIR spectrum of VICWs in archaeological wood. The dramatic intensity decline at both 1730 cm^−1^ and 1234 cm^−1^ indicated the loss of carboxyl groups in glucuronic acids of O-acetyl-(4-O-methylgulcurono) xylan, which was also proved by the absence of signal 16 at 21 ppm and signal 1 at 172 ppm, ascribed to CH_3_-COO- methyl carbon in hemicellulose acetyl groups and the carbonyl of carbohydrates, respectively, in the ^13^C solid state NMR curve of WAW [17,23,24,25] (Figure 4A and Table S2). The loss of carboxyl group of glucuronic acid residues in hemicellulose probably indicates the partial loss of unconjugated ester linkages in lignin-carbohydrate complexes (LCCs) of VICWs in archaeological wood according to a previous report [14]. It is known that the covalent link between the carboxyl group of glucuronic acid residue in hemicelluloses and α-hydroxyl group of the lignin represents one kind of covalent links in LCCs [26,27]. In addition, the weak Py-GC/MS signal 2 (2-butenal), weak signal 3 (2-furan methanol) and weak signal 5 (2-hydroxy-3-methyl-2-cyclopenten-1-one) in the pyrogram of the WAW back up these findings [17,28,29,30,31] (Figure 3B & Table S3). Besides, a band at 1264 cm^−1^, ascribed to aromatic C-O stretching vibrations of methoxyl and phenyl propane units in guaiacol rings of lignin, appeared in the FTIR spectrum of VICWs in WAW (Figure 3I). It resulted from the decrease of peak intensity of band at 1234cm^−1^, as described in our previous research [17]. This breakage of β-O-4 interlinks in lignin was indicated by the decrease in the intensity ratio of signal 10 and signal 11 at 75 ppm and 72 ppm, ascribed to C-OH in β-O-4 linked side chain of lignin and C2,3,5 in carbohydrates in the ^13^C solid state NMR curves of WAW (Figure 4A). The degradation of the aromatic skeletons of syringyl units in lignin was also proven by the decrease in the ratio of intensity between signal 11 and signal 12, that belong to short-chain in syringyl lignin and long-chain in syringyl lignin respectively, as illustrated by the py-GC/MS (Figure 4B). In addition, the relative intensity ratios of bands presented in Figure 3J, were further analyzed by ANOVA. The intensity ratios (I1502/I1730, I1502/I1370 and I1318/I1334) were significantly increased in archaeological wood compared to the RW (*p* < 0.05). The relative intensity ratio for FTIR peaks at 1592 cm^−1^ and 1508cm^−1^ in the spectra of archaeological wood increased to 1.59, while the value of the ratio for the RW was 1.45. This probably reflects the increase of C=O content in lignin after the deterioration. In sum, these results indicate that during the long-term deterioration, the degradation of polysaccharides particularly hemicelluloses was more pronounced than that of lignin, as also proven by the much higher relative content of lignin in the WAW (Table 1). Moreover, cellulose in the amorphous domain was probably degraded more seriously than the crystalline cellulose, which is discussed further in Section 2.2.2.

Spectral images of cell wall components of VICWs in WAW and RW were generated using the absorbance bands at 1592 cm^−1^ assigned to the aromatic skeletal vibrations together with C=O stretch in lignin, 1730 cm^−1^, 1502 cm^−1^ and 1370 cm^−1^, respectively (Figure 3). For the VICWs in WAW, the intensities of each pixel in the spectral images generated using the band 1730 cm^−1^ showed a large decrease, whereas the spectral images obtained using the other bands only revealed slight decreases, in comparison to the RW. This indicated that the degradation of C=O stretch in the glucuronic acid of O-acetyl-(4-O-methylgulcurono) xylan occurred uniformly in VICWs in WAW and that both cellulose and lignin were also degraded, which was in agreement with the ^13^C-NMR and Py-GC/MS results (Figure 4).

**Figure 5:**
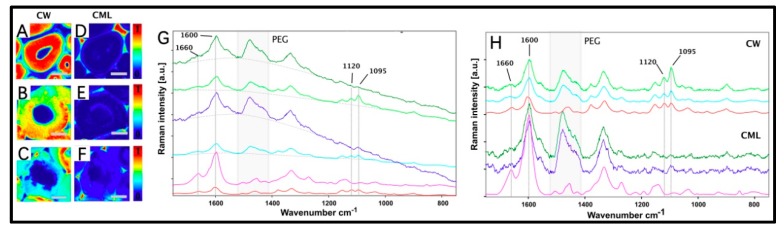
VCA endmember Raman images of the secondary cell wall (CW) endmembers (**A**–**C**) and compound middle lamella (CML) endmembers (**D**–**F**) in RW (**A**,**D**), VICWs in WAW (**B**,**E**) and decayed cell walls in WAW (**C**,**F**). Scale bar = 5 µm. Endmember Raman spectra of specimens without (**G**) and after (**H**) the baseline correction. CW of the RW (the red line), CW of the VICWs in WAW (the cyan line), CW of the decayed cell walls in WAW (the lime line), CML of the RW (the violet line), CML of the VICWs in WAW (the blue line), CML of the decayed cell walls in WAW (the green line).

Raman imaging provided a comprehensive spatially resolved analysis of the wood constituents. As shown in Figure 5B,E, VICWs in WAW were intact. Whereas, cavities, which were probably generated by erosion bacteria or/and soft-rot fungi, were displayed in cell walls of WAW (Figure 5C,F).

The most prominent observation in the endmember spectra of WAW was the drastic increase in fluorescence background. In general, quinoid and carbonyl structural elements in lignin endows the fluorescent properties of wood cell walls [32]. Lähdetie et al. discovered that lignin covalently bonded to the other wood cell wall components exhibited relatively low fluorescence, whereas isolated or chemically treated lignin possessed a flexible conformation that foster fluorescence [33]. Based on their analysis, we interpreted the observed drastic increase of fluorescence background of WAW in the following ways: Firstly, lignin in WAW appears to be less bound to the carbohydrate matrix, enabling a flexible conformation, which was further supported by the severe degradation of hemicelluloses proven by FTIR imaging (Figure 3). Secondly, the structure of lignin was partly altered similar to extracted lignin. Moreover, the relative content of lignin of VICWs in WAW was 21.2% higher than that of RW. These three factors contributed to the drastic increase of the overall Raman background of both VICWs and decayed cell walls (Figure 5G). This also indirectly proved that the increase of relative lignin content is accompanied with the deterioration process and/or the presence of new strong-fluorescent chromophores in lignin [34,35]. Stunningly, decayed and VICWs in WAW revealed the same grade of fluorescence.

For a comparison of the individual bands of cell walls in WAW and in RW the baseline corrected VCA endmember spectra are present in Figure 5H. It should be emphasized that the Raman region from 1420 cm^−1^ to 1510 cm^−1^ was not taken into account due to the influence of the embedding material PEG. The lignin specific band at 1660 cm^−1^, attributed to the conjugated C=C stretching of coniferyl alcohol or the C=O stretch of coniferaldehyde [14,36,37] (Table S4), exhibited substantial differences. CW and CML spectra of WAW revealed decrease of this respective band. Recently, Prats Mateu et al. showed that a decrease of the 1660 cm^−1^ band could be directly related to the polymerization or degradation of monolignols in lignin [36]. Furthermore, the Raman band at 1660 cm^−1^ decreased at the same degree for both visually intact and decayed cell walls in WAW, compared to RW. Additionally, the comparative analysis showed that there was no band broadening, evolvement of new shoulders, or any band shifts between the visually intact and decayed cell walls in WAW (Figure 5H). This indicated that besides the microbial degradation, abiotic decay such as acidic deterioration might also be involved in this specific WAW specimen, since there was no obvious evidence of microbial degradation.

#### 2.2.2. Cellulose Crystallite Structure

**Figure 6:**
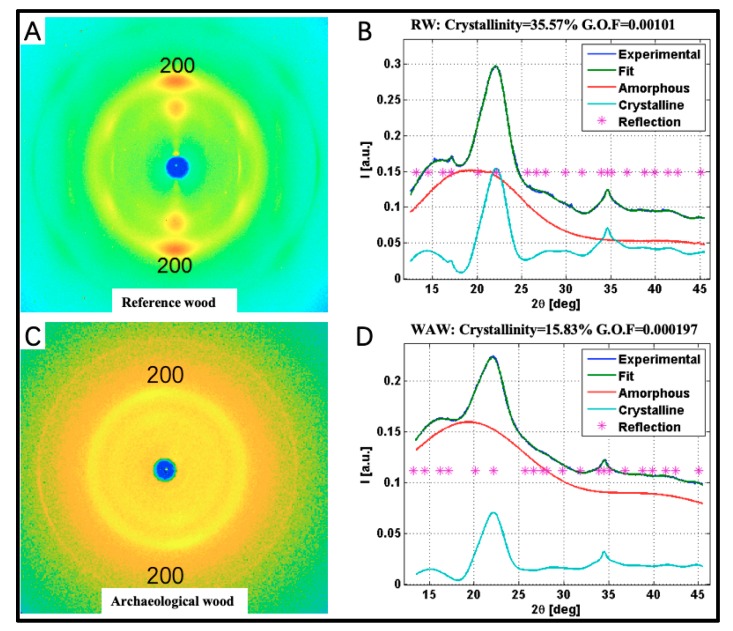
2D scattering pattern from RW (**A**), Archaeological wood (**C**) and corresponding crystallinity fit images (**B**,**D**).

Cell walls of WAW were further studied by WAXS [38] regarding changes of cellulose crystallite structure (Figure 6) after the long-term deterioration. In our setup the analysis integrates a multitude of cells and is therefore not visually intact cells specific but involves also strongly decayed cell walls. Possible alterations of cellulose crystallinity and of cellulose crystallite dimension, which means microfibril diameter, are of importance as they affect the mechanical properties of cell walls [15]. The average relative crystallinities of VICWs in WAW and RW were 17.0% and 36.7% with the standard deviations (between the replicas) 1.4%-units and 1.3%-units, respectively.

A similar decrease of relative crystallinity of WAW was also reported for samples of the Swedish warship Vasa [5]. The average crystallite widths were 30.8 Å and 32.5 Å, with standard deviations of 1.0 Å and 0.2 Å between the replicas, respectively. This decrease in average crystallite width is only minor and not significant and hence one cannot conclude a degradation of crystalline cellulose on the surface of cellulose crystallites.

#### 2.2.3. Porosity

**Figure 7:**
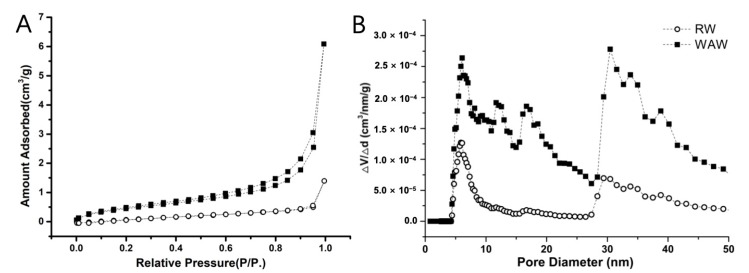
(**A**) Nitrogen adsorption–desorption isotherms and (**B**) corresponding pore size distributions of archaeological wood (the solid square) and the RW (the open dot).

The mesopores in cell walls are also of relevance for the mechanical properties. Hence, we applied nitrogen adsorption to study the change of porous structure of WAW after the long-term deterioration. As shown in Figure 7A, WAW had a higher adsorbed amount than RW at each relative pressure. The isotherms of *Hopea* were classified between type II and type IV according to IUPAC classification [39], suggesting the existence of mesopores and a certain amount of macropores as described by previous researchers [40]. The essential multilayer adsorption process characterizing the mesopore (2 nm < pore diameter < 50 nm) structure could be indicated by the formation of hysteresis loops at a certain relative pressure. The loops belonged to type H3 with slit-shaped pores in this research (Figure 7B).

For WAW specimens, new pore peaks appeared at 8, 9, 11 and 18 nm. Peaks of WAW at 6, 16, 30, 33 and 38 nm had much higher intensities than those of RW. Mesopores with these diameters are known to be present in wood cell walls or in pit membranes [40]. The total pore volumes (V_total_) of WAW and RW were calculated by the cumulative adsorption pore volume using the BJH method to be 0.01 cm^3^/g and 0.003 cm^3^/g, respectively. Specific surface area (S_BET_) of the WAW was 1.836 cm^2^/g while the value was 0.743 cm^2^/g for RW, showing an increase of 147%. These results indicate that more mesopores were generated in cell walls of WAW, which facilitated the penetration of deterioration factors in wood cell walls as well as the loss of degradation products. Furthermore, the mesopores decrease the density of wood cell walls, which affects their mechanical performance of wood cell walls.

### 2.3. Effect of Structure Deterioration on Micromechanics of VICWs in WAW

**Figure 8:**
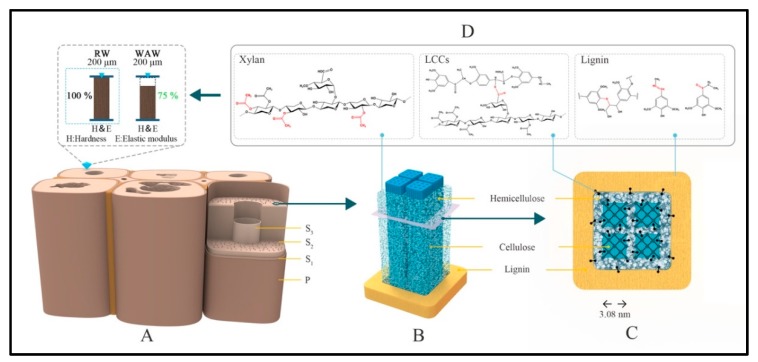
The diagram of elucidating the effect of polysaccharides and lignin deterioration in cell wall on micromechanics of WAW. (**A**) Scheme of S_2_ layer of WAW cell wall and the nanoindentation. (**B**) Microfibril structure in S_2_ layer of WAW. (**C**) The cross section of polysaccharides-lignin structure. (**D**) The main chemical structure of deteriorated chemical functional group in VICWs in WAW.

As natural bio-macromolecular materials, the mechanical properties of wood highly depend on the chemical composition of the cell wall polymers i.e., lignin and polysaccharides including cellulose and hemicelluloses [41]. Among the cell wall layers, secondary cell walls provide most of the mechanical stability to the wood (Figure 8A) due to the parallel alignment of the strong cellulose fibrils, embedded in a matrix of hemicelluloses and lignin [42,43] (Figure 8B,C). Lignin functions as reinforcing agent, reducing the risk of cell wall buckling under compressive load [41,42] (Figure 8B,C). Hemicelluloses typically consist of a backbone and side branches that connect to cellulose and lignin [41] (Figure 8C,D). In particular hemicelluloses are degraded more easily than the other main components of the cell wall [17]. Since stresses are transferred from the matrix polymers to the stiff cellulose fibrils upon mechanical loading [41], degradation of the lignin and cellulose as well as hemicellulose impacts the mechanical properties of the wood cell wall.

Our study showed that even in VICWs in WAW, wood cell wall components including hemicelluloses, cellulose and lignin are substantially altered. For hemicelluloses, carboxyl of side chains were partly removed, and a 68% loss of xylose in xylan was observed (Figure 8C,D). Additional cellulose deterioration was revealed besides the decay of hemicelluloses. The cellulose in the amorphous domain, which was degraded more seriously than the crystalline cellulose, together with the degradation of glucose in hemicelluloses, sum up to a 26% decrease of glucose in cell walls of WAW.

Due to the deterioration of the polysaccharides in WAW, the relative content of lignin increased to 55.6%. Furthermore, the lignin was found to be slightly altered in VICWs of WAW. We propose that lignin has experienced a partial loss of unconjugated ester linkages in LCCs and the reduction of the conjugated C=C of coniferyl alconol/ the C=O of coniferaldehyde loosened the connection between lignin and hemicellulose. The degradation of wood cell wall components further led to the decrease of relative crystallinity of WAW, and the formation of new mesopores in the wood cell wall, resulting presumably in a lower cell wall density of WAW after the long-term deterioration.

The long-term activity of bacteria and perhaps fungi might increase the porosity and permeability of the WAW [44]. In consequence, the mechanical properties of S_2_ layer of VICWs in WAW were decreased by more than 25% compared to cell walls in RW.

## 3. Conclusions

VICWs of WAW possessing an unaltered morphological structure, exhibit a more than 25% decrease in hardness and indentation modulus, compared to RW, which was examined by nanoindentation without embedding. Results indicated that the reduction of mechanical performance of the cell walls was caused by the changes of composition and the porosity after the long-term deterioration. Cell wall features of VICWs in WAW showed the cleavage of carboxyl in side chains of xylan, a severe loss of sugar components in polysaccharides, the partial breakage of β-O-4 interlinks in lignin. These further led to a severe decrease in crystallinity and a slight reduction of cellulose crystallite dimension as well as higher amount of mesopores in cell walls. These cell wall changes contributed to the decrease of mechanical properties of VICWs in WAW.

This study on the effect of structural and chemical deterioration on the micromechanics of VICWs in WAW provides important knowledge for preservation and conservation of waterlogged archaeological wooden artifacts. Furthermore, it may lead to a better understanding of the underlying structure-mechanics relationship of WAW for various deterioration states in our future research. However, not all applied methods allowed for examining the single cell wall, such as ^13^C-solid state NMR, WAXS, etc., which needs to be considered, when focusing on VICWs of waterlogged archaeological wooden artifacts.

## 4. Materials and Methods

### 4.1. Materials

The “Xiaobaijiao No.1” shipwreck is located in Yushan Island, Ningbo City, China (Figure S3). A WAW specimen with dimensions of 40 mm × 40 mm × 15 mm in the radial (R), tangential (T), and longitudinal (L) directions, respectively, was selected from a bottom shell plank of the shipwreck. It was identified as *Hopea* sp. according to its wood anatomy features. The MWC was measured to be 264 ± 81% [45]. According to the MWC criteria, the global deterioration state of WAW was classified into the moderately-deteriorated wood (MWC: 185–400%) [11,12]. Recent *Hopea* wood was chosen from a well recorded xylarium specimen of Wood Collection of Chinese Academy of Forestry as RW.

### 4.2. Methods

#### 4.2.1. SEM

**Figure 9:**
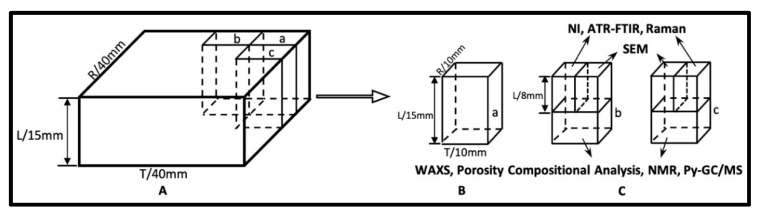
Schematic pictures indicating (**A**) the locations of testing specimens, and the preparations of testing specimens for measurements of (**B**) WAXS and porosity, (**C**) nanoindentation (NI), compositional analysis, ATR-FTIR imaging, Raman imaging, SEM, ^13^C-NMR and Py-GC/MS. Three small wood blocks were named as a, b and c, respectively.

Cross sections of wood samples (Figure 9C) were prepared using microtome (Leica M2255, Leica, Nussloch, Germany). The WAW samples were embedded with polyethylene glycol (PEG) 2000 (Average molecular mass: 1900–2200) during cutting with microtome and then the PEG was dissolved under flow water. All samples were dried, mounted on aluminum stubs, sputter-coated with Platinum, and examined by field emission scanning electron microscopy (Quanta 200F FEI, Thermo Fisher Scientific, Waltham, MA, USA).

#### 4.2.2. Nanoindentation Measurements

Three replicate specimens (0.8 mm (R) × 0.8 mm (T) × 6 mm (L)) were selected randomly from WAW and RW, respectively, (Figure 9C). Specimens were prepared with a diamond knife and then stored in a chamber kept at 20 °C and 65 RH (relative humidity) % for at least one week before testing. Atomic force microscopy (AFM) measurements were performed firstly to determine the position of the indents, to avoid edge effects [18,46]. Nanoindentation of selected positions in wood specimens was then performed by a nanoindenter (Triboindenter TI-900, Hysitron, Eden Prairie, MN, USA) with a peak force of 200 µN at a loading rate of 40 µN/s, which was held for 2s before unloading. At least 30 indentations were made on the transverse sections. The indentation modulus (Figure S4) and the hardness were obtained according to previous study [20,47].

#### 4.2.3. ATR-FTIR Imaging

Dried specimens were conditioned at 12% RH for at least 7 days prior to the measurement. Tangential sections with the thickness of 1 mm were prepared. Measurement was performed with a Spotlight 400 FTIR microscope (Spectrum, Waltham, MA, USA) equipped with a germanium crystal diamond reflection accessory, which provides a high pixel resolution of 1.56 × 1.56 µm^2^. More than 25 points in each full-spectral FTIR absorbance 2D image were randomly selected (Figure S5C,F) with the help of related 3D images (Figure S5B,E). Average spectra of the selected points were then processed by Origin 2017 (OriginLab Corporation, Northampton, MA, USA). Baseline correction was applied according to previous publications [17,47]. Principal component analysis (PCA) was used to identify the relevant spectral bands that account for the degradation of WAW [17,48] using SIMCA 14.1 (Umetrics, Umeå, Sweden).

#### 4.2.4. Confocal Raman Imaging

Specimens were prepared following the protocol of our previous study [49]. Cross sections with the thickness of 10 μm were cut from the specimens (Figure 9C) using a microtome (RM 2255, Leica, Wetzlar, Germany). The WAW was embedded in PEG 2000 prior to cutting. Tests were performed with a confocal Raman microscope (Renishaw InVia, Wotton-under-Edge, England) using the helium-neon laser at the wavelength of 633 nm, an oil immersion objective (Nikon, Tokyo, Japan, 100×, NA = 1.3, 0.17 mm coverslip corrected) and an 1800 mm^−1^ grating. As mapping parameters an integration time of 3.5 s and a step width of 300 nm were used. For Vertex Component Analysis (VCA) the map data were exported into CytoSpec. All the recorded spectra were assigned to each of the endmembers in dependence of their similarity [50]. 3–8 wood cells were selected for each specimen.

#### 4.2.5. Compositional Analysis

The structural carbohydrates and total lignin content of a wood specimen (Figure 9C) were determined following the standard procedure of the National Renewable Energy Laboratory (NREL, Golden, CO, USA) protocol [20,51].

#### 4.2.6. ^13^C NMR Spectra and Py-GC/MS Measurements

Wood powder of freeze-dried archaeological wood (Figure 9C) and RW were prepared by a freeze-grinding treatment using an EFM Freezer Mill 6770 (SPEX SamplePrep, Metuchen, NJ, USA). Solid state ^13^C-NMR spectra were recorded with an Avance III 400 WB NMR spectrometer (Bruker, Billerica, MA, USA) operating at 100.62 MHz. A sample spinning speed of 7 kHz was used. ^13^C-NMR experiments were performed with a 2 ms contact time, a 33 ms acquisition time and a 5 s relaxation delay. Chemical shift values were measured with respect to glycine as a reference with the carbonyl signal set at 38.5 ppm. Py-GC/MS measurements were conducted using a pyrolyser (EGA/PY-3030D, Frontier Laboratories Ltd., Fukushima, Japan) directly attached to gas chromatography/mass spectrometry (QP2010-Ultra, Shimadzu, Duisburg, Germany). A pyrolysis temperature of 550 °C was used. The chromatographic separation of the volatile products was performed using a capillary column (30.0 m × 0.25 mm × 0.25 µm, length × diameter × thickness) (DB-5MS, Agilent, Carpinteria, CA, USA). The temperature of the chromatographic column was progressively increased as reported in our previously published paper [17]. Peak identification was carried out with the NIST mass spectral library and according to the literature [28,52].

#### 4.2.7. Nitrogen Adsorption

Small wood sticks of WAW (Figure 9B) and RW were supercritically dried using a critical point drier (EM CPD300, Leica, Wetzlar, Germany). Nitrogen adsorption tests were carried out using a surface area and pore-size analyzer (AutosorbiQ, Quantachrome, Boynton Beach, FL, USA) at 77 K. Before the adsorption measurements, wood samples were degassed at 80 °C for 8 h under a high vacuum (<10^−5^ Pa). The DFT method was applied to determine the pore size distribution when the mesopore structures of WAW and RW were analyzed.

#### 4.2.8. WAXS

To determine the average width of cellulose crystallites and the relative fraction of crystalline cellulose (i.e., relative crystallinities), WAXS measurements were conducted in perpendicular transmission geometry for five replicates of 1-mm-thick pieces cut from the WAW block (Figure 9B) and three replicates of the corresponding RW samples. X-rays were generated by a conventional Cu-anode X-ray tube (36 kV, 25 mA). Cu-Kα radiation (wavelength is 0.154 nm) was selected by a Montel monochromator. A MAR345 image plate (marXperts, Norderstedt, Germany) was used as detector. The absorption corrections were enabled by using a semitransparent beam stop. The scattering angle range was calibrated with lanthanum hexaboride and the instrumental broadening was determined to be 0.34 degrees. To determine the average width of cellulose crystallites, a 40-degree-wide sector around the cellulose reflection 200 in the WAXS pattern was integrated, and to determine the crystallinity, 180-degree-sector was integrated. From these patterns, the cellulose crystallite widths and the relative crystallinities were determined as explained in our previous study [5]. It should be noted, that only for the RW samples, these analysis could be done using the experimentally measured and verified sulfate lignin amorphous model [38], whereas for all the WAW samples this amorphous model was not valid and thus for the WAW samples, a computational amorphous model created by two wide Gaussians was used.

#### 4.2.9. Data Analysis

One-way statistical analysis of variance (ANOVA) was applied on the data to statistically evaluate WAW and RW regarding elastic modulus and hardness properties of cell walls, ratios of peak intensities calculated from average absorbance of ATR-FTIR absorbance imaging using SPSS 26.0 (IBM Corp., Armonk, NY, USA). PCA analysis was applied with SIMCA program 14.1 (Umetrics, Inc.).

## Figures and Tables

**Table 1:** Chemical compositions of *Hopea* wood.

Sample	Lignin		Carbohydrates	
	Acid-Insoluble Lignin	Acid-Soluble Lignin	Glucose	Xylose
**RW**	34.0%	0.8%	53.0%	12.2%
**WAW**	55.8%	1.0%	39.3%	3.9%

Chemical composition 100% means related to investigated components.

## Supplementary Materials

The following is available online, Figure S1: Light microscopy image of waterlogged archaeological wood. Red arrows marked the VICWs, Figure S2: PCA scores (A) and loading plot of PC1 (B) of Hopea wood samples. R: RW. W: VICWs in archaeological wood, Figure S3: The archaeological site of marine “Xiaobaijiao I” shipwreck. (map source: bzdt.ch.mnr.gov.cn), Figure S4: Examples of load-displacement curves of nanoindentations on the cell walls in RW (the black line) and VICWs in archaeological wood (the red line), Figure S5: Images based on CCD camera (A, D) and pseudo-colour full-spectral FTIR absorbance 3D images (B, E) and related 2D images (C, F) of wood tangential sections; RW (A, B, C) and archaeological Hopea wood (D, E, F), Table S1: Peak assignments for FTIR spectra of wood, Table S2: Resource assignment of solid 13C NMR spectrum of Hopea wood, Table S3: Pyrolysis products of Hopea wood by Py–GC/MS, Table S4: Assignment of Raman bands for main components in hardwood.
