# Supplementary material for: Even Visually Intact Cell Walls in Waterlogged Archaeological Wood Are Chemically Deteriorated and Mechanically Fragile: A Case of a 170 Year-Old Shipwreck
Source: Molecules. 2020 Mar 3;25(5):1113. doi: 10.3390/molecules25051113 (PMC7179126; doi:10.3390/molecules25051113)
Supplement: Supplementary file 1 [file molecules-25-01113-s001.pdf]

Supplementary material

Even Visually Intact Cell Walls in Waterlogged Archaeological Wood are Chemically Deteriorated and Mechanically Fragile: A Case of a 170-year-old Shipwreck

Liuyang Han[1,2], Xingling Tian[3], Tobias Keplinger[4,5], Haibin Zhou[6], Ren Li[1,2], Kirsi Svedström[7], Ingo Burgert[4,5], Yafang Yin[1,2] and Juan Guo[*1,2]

[1] Department of Wood Anatomy and Utilization, Research Institute of Wood Industry, Chinese Academy of Forestry, Beijing 100091, China;
[2] Wood Collections (WOODPEDIA), Chinese Academy of Forestry, Beijing100091, China;
[3] Heritage Conservation and Restoration Institute, Chinese Academy of Cultural Heritage, Beijing 100029, China;
[4] Wood Materials Science, ETH Zürich, 8093 Zürich, Switzerland;
[5] Laboratory for Cellulose & Wood Materials, EMPA, 8600 Dübendorf, Switzerland;
[6] Pilot Base, Chinese Academy of Forestry, Beijing 102300, China;
[7] Department of Physics, University of Helsinki, FI-00014 Helsinki, Finland
* Correspondence: guojuanchina@126.com; (J.G.)

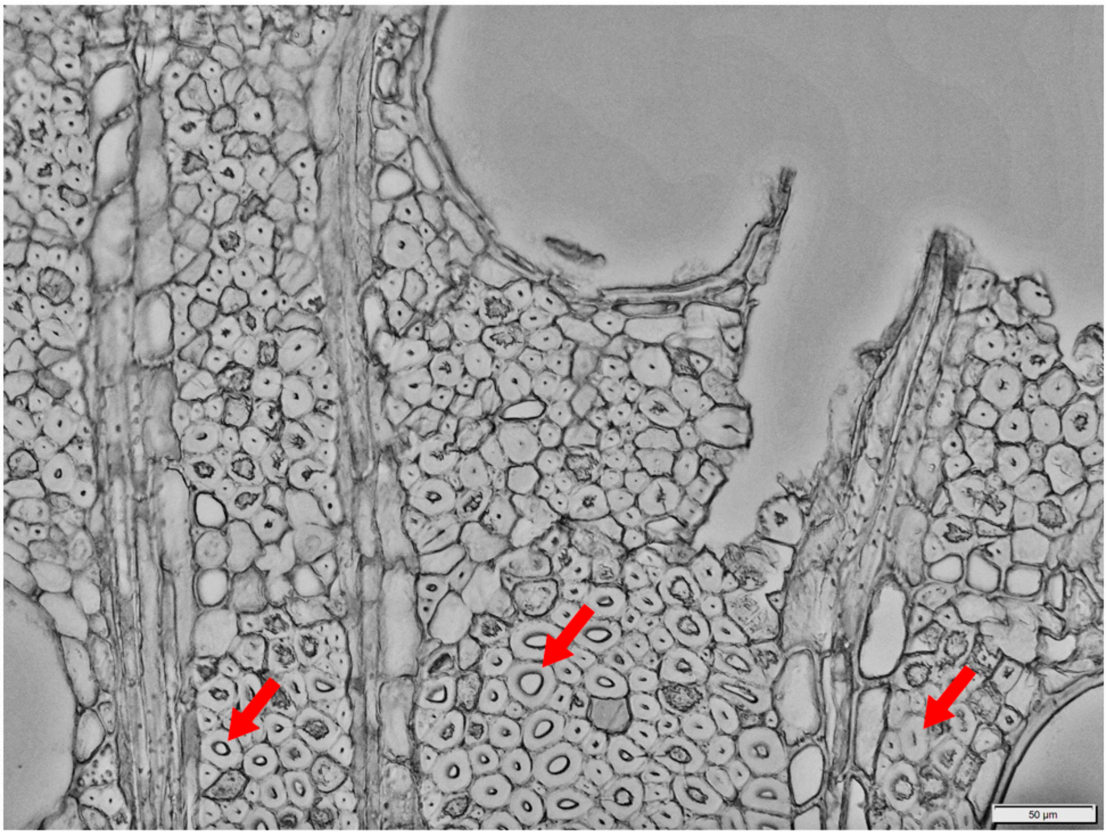

Figure S1. Light microscopy image of waterlogged archaeological wood. Red arrows marked the VICWs.

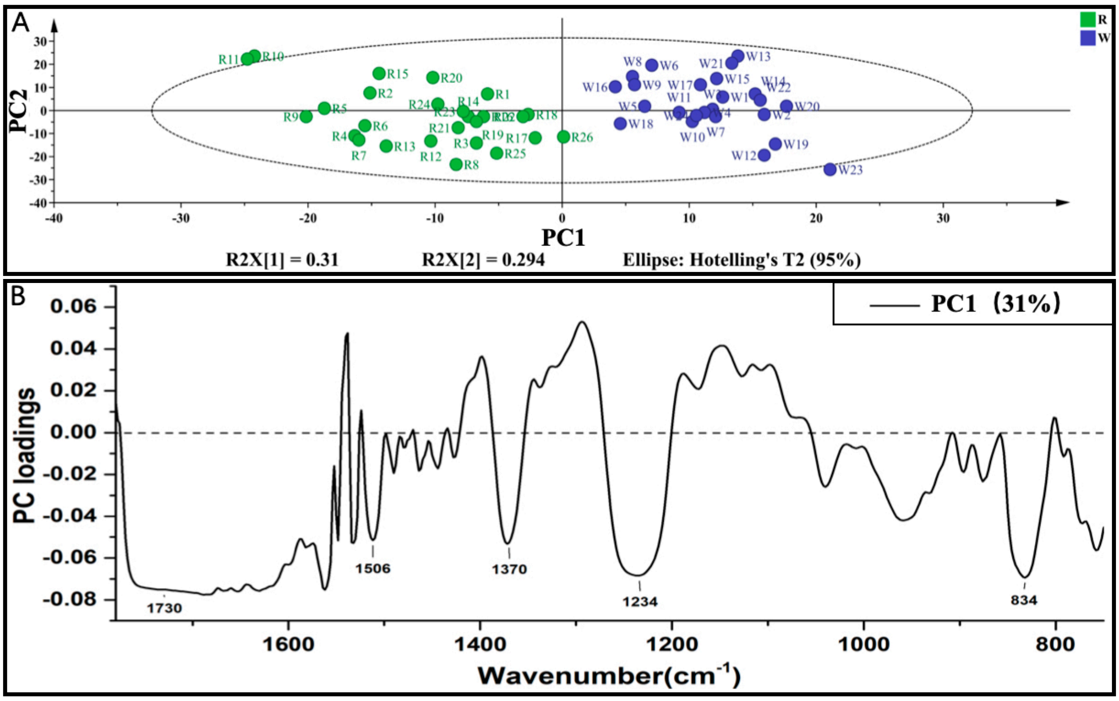

Figure S2. PCA scores (A) and loading plot of PC1 (B) of *Hopea* wood samples. R: RW. W: VICWs in archaeological wood.

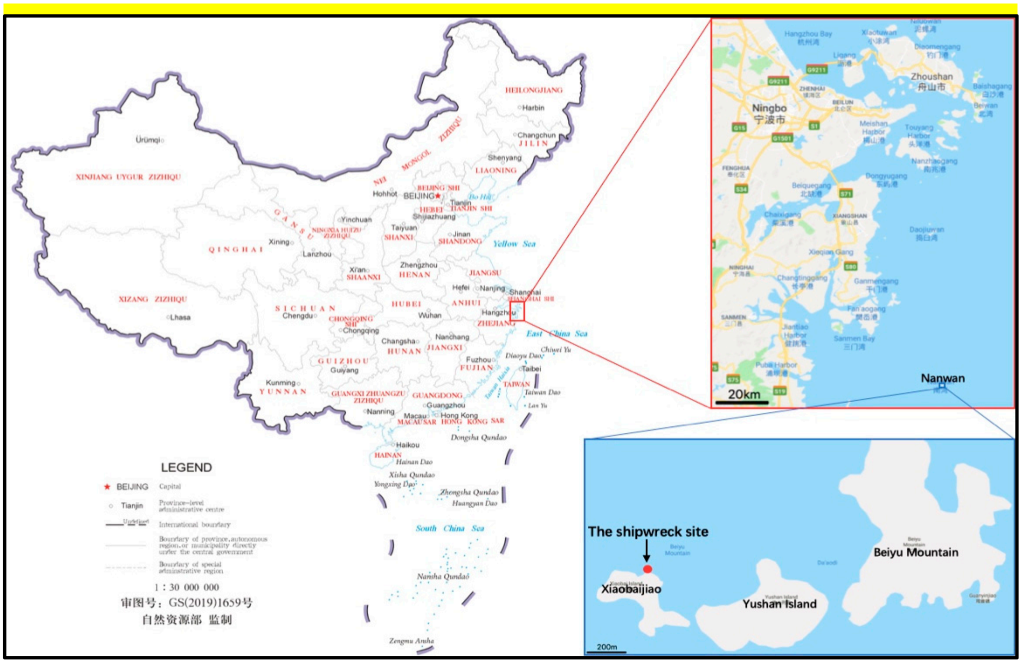

Figure S3. The archaeological site of marine "Xiaobaijiao I" shipwreck. (map source: bzdt.ch.mnr.gov.cn)

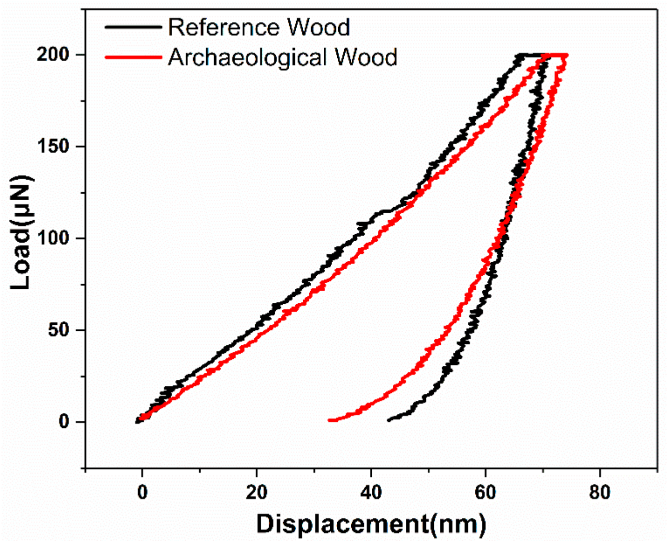

Figure S4. Examples of load-displacement curves of nanoindentations on the cell walls in RW (the black line) and VICWs in archaeological wood (the red line).

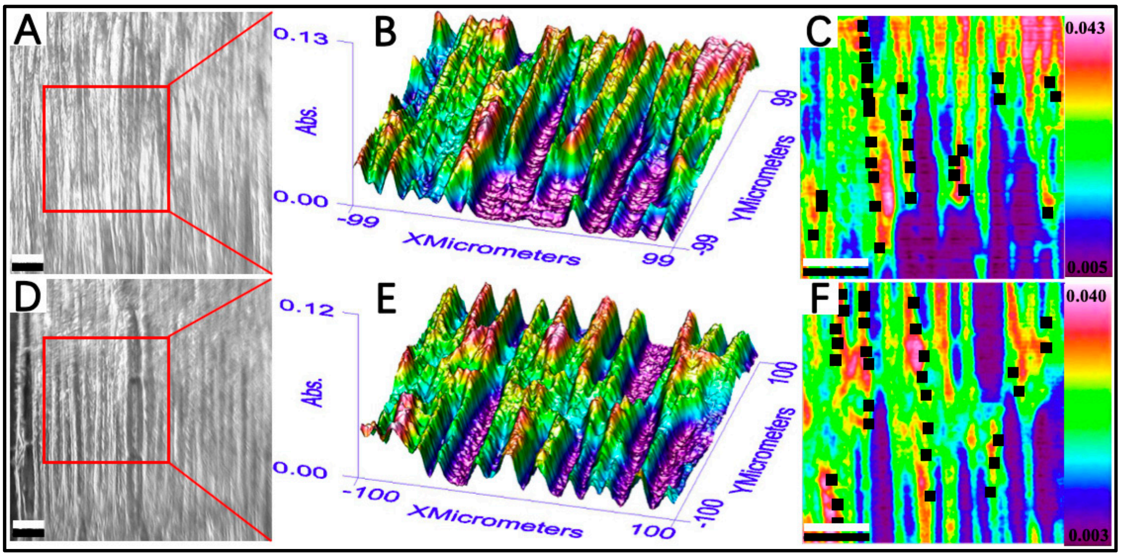

Figure S5. Images based on CCD camera (A, D) and pseudo-colour full-spectral FTIR absorbance 3D images (B, E) and related 2D images (C, F) of wood tangential sections; RW (A, B, C) and archaeological Hopea wood (D, E, F). The intensity of the absorbance is indicated by the change in color. The black solid squares present in Fig. 2C and Fig. 2F indicated the chosen pixel positions in the S_2 layer of wood fibers in specimens. Scale bar = 50 μm.

Table S1. Peak assignments for FTIR spectra of wood [16, 22, 43, 47]

Peak position (cm^{-1})	Peak assignment
**3700~3100**	the hydroxyl stretching region
**1730**	mainly assigned to the C=O stretch in acetyl groups of hemicellulose
**1592**	the relative concentration of aromatic skeletal vibrations, together with C=O stretch in lignin
**1502**	the aromatic skeletal vibration in the lignin (mainly syringyl)
**1462**	the CH_2 symmetric bending on the xylose ring
**1424**	the CH_2 scissoring in cellulose
**1370**	the C-H bending in polysaccharides
**1334**	the O-H in-plane bending of amorphous cellulose
**1318**	the wagging (out of the plane) of the CH_2 groups in crystalline cellulose
**1264**	the aromatic C-O stretching vibrations of methoxyl and phenyl propane units in guaiacol rings of lignin
**1234**	mainly assigned to the C-O stretching in the O=C-O group of side chains in hemicellulose
**1220**	the aromatic C-O stretching vibrations in syringol rings of lignin
**1160**	the characteristic of asymmetric bridge C-O-C stretch mode in polysaccharides
**897**	β-anomers or β-linked glucose polymer
**834**	the C–H out of plane deformation in position 2 and 6 of syringyl unit in lignin
**810**	equatorially aligned hydrogen on the C_2 atom in the mannose residue in glucomannan

Table S2. Resource assignment of solid ^{13}C NMR spectrum of *Hopea* wood [17, 23-25]

Resonance number	Chemical shift (ppm)	Assignments
**1**	173	Carbohydrates: -COO-R, CH_3-COO-
**2**	152.6	Lignins: S_3 (e), S_5 (e)
**3**	147	Lignins: S_3 (ne), S_5 (ne), G_3, G_4
**4**	136	Lignins: S_1 (e), S_4 (e), G_1 (e)
**5**	134	Lignins: S_1 (ne), S_4 (ne), G_1 (e)
**6**	127-116	Lignins: G_6, G_5, S_2, S_6
**7**	105	Carbohydrates: C_1
**8**	88.7	Carbohydrates: C_4
**9**	84	Lignins: C_β in β-O-4 linked side chain; Carbohydrates: C_4
**10**	75	Lignins: C_α -OH in β -O-4 linked side chain of lignins; Carbohydrates: $C_{2,3,5}$
**11**	72	Carbohydrates: $C_{2,3,5}$
**12**	65	Carbohydrates: C_6
**13**	62	Lignins: C_γ
**14**	56	Lignin: OCH_3
**15**	33	long-chain CH_2 included in resin acids, residues of microbial biomass, or condensed tannin
**16**	21	Polysaccharides: CH_3-COO- [methyl carbon in hemicellulose acetyl groups]

S: syringyl (aromatic unit with two methoxyl groups); G: guaiacyl (aromatic unit with only one methoxyl); ne: in non-etherified arylglycerol β-aryl ethers; e: in etherified arylglycerol β-aryl ethers.

Table S3. Pyrolysis products of *Hopea* wood by Py–GC/MS [17, 28-31].

No.	Retention time (min)	Name of compound	Origin	Category
**1**	2.2	acetic acid	holocellulose	
**2**	2.4	2-butenal	holocellulose	
**3**	5.5	2-furan methanol	holocellulose	
**4**	9.2	phenol	p-hydroxyphenyl lignin	others
**5**	9.5	2-hydroxy-3-methyl-2-cyclopenten-1-one	holocellulose	
**6**	10.0	o-cresol	p-hydroxyphenyl lignin	others
**7**	10.4	m (or p)-cresol	p-hydroxyphenyl lignin	others
**8**	13.1	syringol	syringyl lignin	short-chain
**9**	14.0	E-isoeugenol	guaiacyl lignin	long-chain
**10**	14.6	5-tert-butyl-1,2,3-benzentriol	lignin	dem
**11**	15.0	4-vinylsyringol	syringyl lignin	short-chain
**12**	15.2	4-allylsyringol	syringyl lignin	long-chain

*dem: demethylated/demthoxylated lignin pyrolysis products.

Table S4. Assignment of Raman bands for main components in hardwood [14, 16, 17].

Wavenumber (cm^{-1})	Assignment	Component
1096	Heavy atom (C-C and C-O) stretching	Cellulose, Glucomannan, Xylan
1121	Heavy atom (C-C and C-O) stretching	Cellulose, Glucomannan, Xylan
1149	Heavy atom (C-C and C-O) stretching plus HCC and HCO bending	Cellulose
1271	Aryl-O of aryl-OH and aryl-O-CH_3; guaiacyl ring (with C=O group) mode	Lignin
1333	Aliphatic O-H bending	Lignin
1600	Aryl ring stretching, symmetric	Lignin
1620	Ring conjugated C=C stretch of coniferaldehyde	Lignin
1660	Ring conjugated C=C stretch of coniferyl alcohol; C=O stretch of coniferaldehyde	Lignin

References

14. Pedersen, N.B.; Gierlinger, N.; Thygesen, L.G. Bacterial and abiotic decay in waterlogged archaeological Picea abies (L.) Karst studied by confocal Raman imaging and ATR-FTIR spectroscopy. Holzforschung 2015, 69(1), 103-112.

16. Guo, J.; Zhou, H.; Stevanic, J. S.; Dong, M.; Yu, M.; Salmén, L.; Yin, Y., Effects of ageing on the cell wall and its hygroscopicity of wood in ancient timber construction. *Wood Science and Technology* **2017**.

17. Guo, J.; Xiao, L.; Han, L.; Wu, H.; Yang, T.; Wu, S.; Yin, Y.; Donaldson, L. A., Deterioration of the cell wall in waterlogged wooden archeological artifacts, 2400 years old. *IAWA Journal* **2019**, 1, 1-25.

22. Simonović, J.; Stevanic, J.; Djikanović, D.; Salmén, L.; Radotić, K., Anisotropy of cell wall polymers in branches of hardwood and softwood: a polarized FTIR study. *Cellulose* **2011,** 18, (6), 1433-1440.

23. Cha, M. Y.; Lee, K. H.; Kim, Y. S., Micromorphological and chemical aspects of archaeological bamboos under long-term waterlogged condition. *International Biodeterioration & Biodegradation* **2014,** 86, 115-121.

24. Bardet, M.; Gerbaud, G.; Giffard, M.; Doan, C.; Hediger, S.; Pape, L. L., 13C high-resolution solid-state NMR for structural elucidation of archaeological woods. *Progress in Nuclear Magnetic Resonance Spectroscopy* **2009,** 55, (3), 199-214.

25. Davis, M. F.; Schroeder, H. R.; Maciel, G. E., Solid-state 13C nuclear magnetic resonance studies of wood decay. I. White rot decay of Colorado blue spruce. Walter de Gruyter,

Berlin/New York: 1994.
28. Faix, O.; Meier, D.; Fortmann, I., Thermal degradation products of wood. Gas chromatographic separation and mass spectrometric characterization of monomeric lignin-derived products. *Holz als Roh- und Werkstoff (Germany, F.R.)* **1991,** 49, (7-8), 299-304.
29. RıÓ, J. C. D.; Speranza, M.; Gutiérrez, A.; MartıÑez, M. J.; MartıÑez, A. T., Lignin attack during eucalypt wood decay by selected basidiomycetes: a Py-GC/MS study. *Journal of Analytical & Applied Pyrolysis* **2002,** 64, (2), 421-431.
30. Tamburini, D.; Łucejko, J. J.; Zborowska, M.; Modugno, F.; Prądzyński, W.; Colombini, M. P., Archaeological wood degradation at the site of Biskupin (Poland): wet chemical analysis and evaluation of specific Py-GC/MS profiles. *Journal of Analytical & Applied Pyrolysis* **2015,** 115, 7-15.
31. Fors, Y.; Nilsson, T.; Risberg, E. D.; Sandström, M.; Torssander, P., Sulfur accumulation in pinewood (Pinus sylvestris) induced by bacteria in a simulated seabed environment: Implications for marine archaeological wood and fossil fuels. *International Biodeterioration & Biodegradation* **2008,** 62, (4), 336-347.
36. Prats-Mateu, B.; Bock, P.; Schroffenegger, M.; Toca-Herrera, J. L.; Gierlinger, N., Following laser induced changes of plant phenylpropanoids by Raman microscopy. *Scientific Reports* **2018,** 8, (1).
37. Gierlinger, N.; Schwanninger, M., Chemical imaging of poplar wood cell walls by confocal Raman microscopy. *Plant Physiol* **2006,** 140, (4), 1246-54.
43. Salmén, L.; Stevanic, J. S.; Olsson, A.-M., Contribution of lignin to the strength properties in wood fibres studied by dynamic FTIR spectroscopy and dynamic mechanical analysis (DMA). *Holzforschung* **2016,** 70, (12).
47. Yin, Y.; Berglund, L.; Salmén, L., Effect of Steam Treatment on the Properties of Wood Cell Walls. *Biomacromolecules* **2011,** 12, (1), 194.
